# Supplementary material for: The Effect of High Voltage Electrical Discharge on the Physicochemical Properties and the Microbiological Safety of Rose Hip Nectars
Source: Foods. 2022 Feb 23;11(5):651. doi: 10.3390/foods11050651 (PMC8909758; doi:10.3390/foods11050651)
Supplement: Supplementary file 1 [file foods-11-00651-s001.zip › foods-1591997-supplementary.pdf]

Table S1. Pearson correlation coefficients between physicochemical parameters, antioxidant activity (ABTS and DPPH), and colour parameters (L* and a*) of rose hip nectar treated with different HVED frequencies and time ($p < 0.05$).

	**pH**	**Electrical conduct.**	**Ascorbic acid**	**TPC**	**Flavan-3-ols**	**Flavonols**	**ABTS**	**DPPH**	**L***	**a***
pH	1									
Electrical conduct.	**-0.842**	1								
Ascorbic acid	-0.512	0.537	1							
TPC	-0.165	0.275	0.511	1						
Flavan-3-ols	-0.468	0.514	0.539	0.258	1					
Flavonols	-0.560	0.488	0.494	-0.007	0.179	1				
ABTS	0.236	0.032	-0.423	-0.248	-0.106	-0.581	1			
DPPH	0.194	-0.196	-0.197	-0.138	-0.478	-0.571	0.634	1		
L*	0.679	-0.600	-0.455	-0.186	-0.322	-0.804	0.696	0.686	1	
a*	0.678	-0.600	-0.456	-0.220	-0.364	-0.752	0.679	0.691	**0.955**	1

Table S2. The results of the post-hoc Tukey test applied on physicochemical parameters, antioxidant activity (ABTS and DPPH), and colour parameters (L* and a*) of rose hip nectar treated with different HVED frequencies (50 and 100 Hz). The time of treatment was 10, 15, and 20 minutes.

F (Hz)	Time (min)	Electrical conduct.	Ascorbic acid	TPC	Flavan-3-ols	Flavonols	AA		Colour parameter	
							ABTS	DPPH	L*	a*
	10									
0		b	b	b	a	b	a	a	a	a
50		b	a	a	a	a	c	b	b	b
100		a	a	a	a	a	b	a	b	b
	15									
0		c	a	a	b	a	a	a	a	a
50		b	a	b	a,b	a	c	b	b	b
100		a	a	a	a	a	b	b	b	b
	20									
0		c	c	c	a	b	a	a	a	a
50		b	b	a	a	a	b	b	b	b
100		a	a	b	a	a	b	b	b	b

Differences in the same column are marked with different letters (post-hoc Tukey test $p \leq 0.05$).
Flavan-3-ols - as a (+)-catechin.
Flavonols - as a quercetin-3-galactoside.
GAE - gallic acid equivalent.
TE - Trolox equivalent.

Table S3. The results of two-factor ANOVA analysis of physicochemical parameters, antioxidant activity (ABTS and DPPH), and colour parameters (L* and a*) of HVED treated rose hip nectar.

	**Electrical conduct.**		**Ascorbic acid**		**TPC**		**Flavan-3-ols**		**Flavonols**		**ABTS**		**DPPH**		**L***		**a***	
	F	P	F	P	F	P	F	P	F	P	F	P	F	P	F	P	F	P
Freq.	316.292	0.000	4.539	0.077	0.157	0.857	7.948	0.030	0.033	0.862	10.105	0.005	2.632	0.126	2.306	0.155	1.071	0.383
Time	121.179	0.000	27.181	0.001	28.317	0.000	19.700	0.002	0.018	0.982	8.427	0.009	0.523	0.610	2.184	0.169	0.154	0.859
Inter.	39.274	0.000	4.763	0.058	4.197	0.034	21.242	0.002	1.122	0.386	6.301	0.011	0.923	0.492	0.773	0.569	0.916	0.495

ANOVA analysis with P-value ≤ 0.05 or F value ≥ $F_{critical}$ are statistically significant
F - F value
P - P value.

Table S4. Pearson correlation coefficients between physicochemical parameters and antioxidant activity (ABTS and DPPH) of different formulated rose hip nectars ($p < 0.05$).

**Parameter**	**Electrical conduct.**	**Ascorbic acid**	**TPC**	**Flavan-3-ols**	**Flavonols**	**ABTS**	**DPPH**
Electrical conduct.	1						
Ascorbic acid	0.268	1					
TPC	0.326	0.663	1				
Flavan-3-ols	-0.650	0.316	-0.052	1			
Flavonols	-0.347	0.584	0.547	0.276	1		
ABTS	-0.544	0.584	0.513	0.691	0.835	1	
DPPH	-0.355	0.688	0.616	0.633	0.779	**0.937**	1

Table S5. Pearson correlation coefficient between colour parameters (L*, a*, b*, C* and h°) of different formulated rose hip nectars ($p < 0.05$).

Parameter	L*	a*	b*	C*	h°
L*	1				
a*	-0.700	1			
b*	**0.987**	-0.697	1		
C*	**0.938**	-0.533	**0.925**	1	
h°	**0.906**	**-0.921**	**0.908**	0.794	1

Table S6. The average counts of aerobic mesophilic bacteria (AMB) and *Enterobacteriaceae* (EB) in the nectars during 12 days of refrigerated storage.

AMB			EB		
**Sample/ Day**	**Microbiol. limit (cfu/mL)**	**Results (cfu/mL)**	**Sample/Day**	**Microbiol. limit (cfu/mL)**	**Results (cfu/mL)**
Day “0“			Day “0“		
N	10^3	<10	N	10	<10
NP	10^3	<10	NP	10	<10
NSP	10^3	<10	NSP	10	<10
NBP 1	10^3	<10	NBP 1	10	<10
NBP 2	10^3	<10	NBP 2	10	<10
NPA	10^3	<10	NPA	10	<10
Day 6			Day 6		
N	10^3	<10	N	10	<10
NP	10^3	<10	NP	10	<10
NSP	10^3	<10	NSP	10	<10
NBP 1	10^3	<10	NBP 1	10	<10
NBP 2	10^3	<10	NBP 2	10	<10
NPA	10^3	<10	NPA	10	<10
Day 12			Day 12		
N	10^3	<10	N	10	<10
NP	10^3	<10	NP	10	<10
NSP	10^3	<10	NSP	10	<10
NBP 1	10^3	<10	NBP 1	10	<10
NBP 2	10^3	<10	NBP 2	10	<10
NPA	10^3	<10	NPA	10	<10

N - nectar without HVED treatment (control)
NP - HVED treated nectar
NSP - HVED treated low-calorie nectar
NBP 1 - nectar prepared from blanched pulp + HVED
NBP 2 - nectar prepared from blanched puree + HVED
NPA - pasteurised nectar.

Table S7. The results of the detection of *Salmonella* spp.(S), *Listeria monocytogenes* (LM), and average counts of *Escherichia coli* (EC) in the nectars during 12 days of refrigerated storage.

	S		LM		EC		
Sample/ Day	Microbiol. limit (cfu/mL)	Results (cfu/mL)	Microbiol. limit (cfu/mL)	Results (cfu/mL)	Sample/ Day	Microbiol. limit (cfu/mL)	Results (cfu/mL)
Day "0"	absence in 25 mL		absence in 25 mL			Day "0"	
N		nd		nd	N	10^2	<10
NP		nd		nd	NP	10^2	<10
NSP		nd		nd	NSP	10^2	<10
NBP 1		nd		nd	NBP 1	10^2	<10
NBP 2		nd		nd	NBP 2	10^2	<10
NPA		nd		nd	NPA	10^2	<10
Day 6	absence in 25 mL		absence in 25 mL			Day 6	
N		nd		nd	N	10^2	<10
NP		nd		nd	NP	10^2	<10
NSP		nd		nd	NSP	10^2	<10
NBP 1		nd		nd	NBP 1	10^2	<10
NBP 2		nd		nd	NBP 2	10^2	<10
NPA		nd		nd	NPA	10^2	<10
Day 12	absence in 25 mL		absence in 25 mL			Day 12	
N		nd		nd	N	10^2	<10
NP		nd		nd	NP	10^2	<10
NSP		nd		nd	NSP	10^2	<10
NBP 1		nd		nd	NBP 1	10^2	<10
NBP 2		nd		nd	NBP 2	10^2	<10
NPA		nd		nd	NPA	10^2	<10

N - nectar without HVED treatment (control)
NP - HVED treated nectar
NSP - HVED treated low-calorie nectar
NBP 1 - nectar prepared from blanched pulp + HVED
NBP 2 - nectar prepared from blanched puree + HVED

NPA - pasteurised nectar.
